# Prognostic Factors for Myositis-Associated Interstitial Lung Disease

**DOI:** 10.1371/journal.pone.0098824

**Published:** 2014-06-06

**Authors:** Tomoyuki Fujisawa, Hironao Hozumi, Masato Kono, Noriyuki Enomoto, Dai Hashimoto, Yutaro Nakamura, Naoki Inui, Koshi Yokomura, Naoki Koshimizu, Mikio Toyoshima, Toshihiro Shirai, Kazumasa Yasuda, Hiroshi Hayakawa, Takafumi Suda

**Affiliations:** 1 Second Division, Department of Internal Medicine, Hamamatsu University School of Medicine, Hamamatsu, Japan; 2 Department of Clinical Pharmacology and Therapeutics, Hamamatsu University School of Medicine, Hamamatsu, Japan; 3 Department of Respiratory Medicine, Seirei Mikatahara General Hospital, Hamamatsu, Japan; 4 Department of Respiratory Medicine, Fujieda Municipal General Hospital, Fujieda, Japan; 5 Department of Respiratory Medicine, Hamamatsu Rosai Hospital, Hamamatsu, Japan; 6 Department of Respiratory Medicine, Shizuoka General Hospital, Shizuoka, Japan; 7 Department of Respiratory Medicine, Iwata City Hospital, Iwata, Japan; 8 Department of Respiratory Medicine, Tenryu Hospital, National Hospital Organization, Hamamatsu, Japan; Keio University School of Medicine, Japan

## Abstract

**Background:**

Interstitial lung disease (ILD) is a common manifestation of polymyositis (PM), dermatomyositis (DM), and clinically amyopathic dermatomyositis (CADM); however, little is known about the factors influencing the prognosis for PM/DM/CADM-associated ILD. (PM/DM/CADM-ILD). The aim of the present study is to assess prognostic factors for PM/DM/CADM-ILD.

**Methods:**

The clinical features and survival of 114 consecutive patients diagnosed with PM/DM/CADM-ILD (39 men and 75 women; median age, 56 years) were analyzed retrospectively.

**Results:**

The study group included 30 PM-associated ILD, 41 DM-associated ILD, and 43 CADM-associated ILD cases. The clinical presentation of ILD was acute/subacute form in 59 patients (51.8%) and chronic form in 55 patients (48.2%). The major pulmonary symptoms were dyspnea, cough, and fever. High-resolution computed tomography frequently revealed ground-glass opacities, traction bronchiectasis, and consolidation. Most of the patients were treated with corticosteroids or corticosteroids in combination with immunosuppressive agents. The all-cause mortality was 27.2%. Acute/subacute form, % forced vital capacity (FVC), age, % of neutrophils in bronchoalveolar lavage (BAL) fluid, and a diagnosis of CADM (vs. PM) were significantly associated with poor outcome in univariate Cox proportional hazards models. Multivariate Cox proportional hazards analysis validated acute/subacute ILD, %FVC, age, and diagnosis of CADM (vs. PM) as significant predictors of overall mortality. Patients with acute/subacute ILD had a much lower survival rate than those with the chronic form (p<0.001). Patients with CADM-ILD had a lower survival rate than those with PM-ILD (p = 0.034).

**Conclusions:**

Acute/subacute form, older age, lower level of FVC and diagnosis of CADM predict poor outcome in PM/DM/CADM-ILD.

## Introduction

The idiopathic inflammatory myopathies (IIMs) are a group of systemic inflammatory diseases that affect skeletal muscles and result in proximal muscle weakness, elevated muscle enzyme levels and extramuscular manifestations such as fever, weight loss and rash [1]. Polymyositis (PM) and dermatomyositis (DM) are two major diseases affecting the muscles, skin, and other organs, including the lungs, in patients with IIMs [1], [2]. Clinically amyopathic dematomyositis (CADM) is a distinct subgroup of DM that causes the typical skin rash of classic DM with little or no evidence of muscular manifestations [3], [4]. Recent studies have revealed that interstitial lung disease (ILD) to be a crucial extramuscular manifestation of PM, DM and CAMD patients that is associated with high mortality [5]–[8]. It is estimated that 20–40% of patients diagnosed with PM/DM/CADM will be afflicted with ILD during the course of their illness [4], [8], [9]. Therefore, evaluation of the presence and severity of ILD is essential to the care of patients with PM/DM/CADM.

Little data is available regarding the details of the clinical course of and prognostic factors for PM/DM/CADM-associated ILD (PM/DM/CADM-ILD). We have previously reported that DM-associated ILD (DM-ILD) is more refractory to corticosteroid therapy and carries a poorer prognosis than PM-associated ILD (PM-ILD) [6]. In terms of CADM, several studies have demonstrated rapidly progressive CADM-associated ILD (CADM-ILD) with poor prognosis [7], [10]–[13]. In addition, our previsous study showed that CADM-ILD included two different forms, acute/subacute and chronic forms, and that mortality was much higher among patients with the acute/subacute form than among those with the chronic form [7]. These reports indicate that there are variations in the clinical course of PM/DM/CADM-ILD, which may complicate appropriate treatment of affected patients. It is essential for clinicians to identify patients in high-risk groups at the time of diagnosis in order to provide better management. The aims of this retrospective study were to assess the clinical characteristics and outcomes of and elucidate prognostic factors for patients with PM/DM/CADM-ILD in a large series of patients.

## Methods

### Patient Selection

The subjects of the study were 114 patients diagnosed with PM (n = 30), DM (n = 41), or CADM (n = 43) with ILD from 1990 through 2012 at Hamamatsu University Hospital and affiliated hospitals in Japan. The diagnosis of PM or DM was based on the criteria of Bohan and Peter criteria [2]: 1) systemic muscle weakness, 2) increased serum muscle enzyme levels, 3) electromyographic (EMG) evidence of myopathic changes, 4) typical histologic findings in muscle biopsies, and/or 5) characteristic dermatologic manifestations of DM. The diagnosis was considered definite, probable, or possible according to the number of criteria fulfilled (at least 4, 3, or 2, respectively, including the dermatologic manifestations for diagnosis of DM), and patients with definite or probable PM/DM were included in the study. CADM was diagnosed when a patient had a skin rash characteristic of DM without clinical evidence of muscle disease and with little or no increase in the serum creatine kinase (CK) level [3], [7], [11], [14]. Most patients visited the pulmonary division because of respiratory symptoms such as shortness of breath, chronic cough, or abnormal findings on chest radiographs; some were first examined by a rheumatologist and then referred to a pulmonologist. This study was approved by the Institutional Review Board of the Hamamatsu University School of Medicine (approval number 25-215). Patient approval or informed consent was waived because the study involved a retrospective review of patient records and images.

### ILD Presentations

ILD was diagnosed on the basis of the presence of radiological abnormalities in combination with respiratory symptoms. Patients were classified as having acute/subacute or chronic ILD according to the criteria that we described in the previous study [7] with slight modification. The acute/subacute form was defined as a rapidly progressive ILD showing deterioration within three months. According to the International Consensus Statement of idiopathic pulmonary fibrosis of the American Thoracic Society (ATS) with modification [15], [16], the deterioration was defined by two or more of the following: 1) symptomatic exacerbation (dyspnea upon exertion); 2) an increase in the severity of parenchymal abnormality on a high-resolution computed tomography (HRCT) scan; and 3) at least one of the following physiological changes: **>**10% decrease in forced vital capacity (FVC) or **>**10 mm Hg decrease in arterial oxygen tension (PaO_2_). The chronic form of ILD was defined as a slowly progressive presentation with gradual deterioration over more than three months. Concomitant onset of myositis and ILD was defined as onset within an interval of three months.

### Data Collection

The patients’ clinical data, including history, treatment, and laboratory findings, were obtained from the medical records from the first encounter that eventually led to a diagnosis of ILD and PM/DM/CADM. Relevant signs and symptoms were also recorded. The following pulmonary function test parameters were assessed: FVC, forced expiratory volume in one second (FEV1.0) and diffusing capacity of the lung for carbon monoxide (DLCO). The bronchoalveolar lavage (BAL) findings were retrospectively reviewed. BAL was performed using three 50-mL aliquots of sterile 0.9% saline, as described previously [7], [17]. The total cell count in BAL fluid was determined using a hemocytometer, and a differential cell count was performed using Giemsa-stained cytocentrifuged preparations. To characterize the phenotype of the lymphocytes in the BAL fluid, flow cytometric analysis was performed in a flow cytometer using mAb OKT4 (anti-CD4; Coulter Electronics), and OKT8 (anti-CD8, Coulter Electronics).

### HRCT Findings

HRCT images taken at the time of PM/DM/CADM-ILD diagnosis were reviewed. These images comprised 1.0–1.5 mm collimation sections at 10 mm intervals. The HRCT images were randomized and reviewed independently by 2 pulmonologists (H.H., M.K.) who were blinded to the clinical data. The images were assessed for the presence of each of the following signs: consolidation, ground glass opacities, traction bronchiectasis, irregular linear opacities, bronchovascular bundle thickening, honeycombing and pleural effusion according to Fleischner criteria [18] with slight modifications. Consolidation was defined as homogeneous increase in pulmonary parenchymal attenuation that obscured the underlying vessels. Ground glass opacities were defined as hazy increased attenuation of the lung, which did not obscure the underlying vessels. Traction bronchiectasis was defined as irregular bronchial dilatation within or around areas with parenchymal abnormality. Irregular linear opacities were defined as elongated line of soft tissue attenuation distinct from interlobular septa and bronchovascular bundles. Bronchovascular bundle thickening were defined as an increase in bronchial wall thickness and diameter of pulmonary artery branches caused by thickened peribronchovascular interstitium. Honeycombing was defined as the appearance of clustered cystic air spaces, typically of comparable diameters on the order of 3 to 10 mm but occasionally as large as 2.5 cm, in the subpleural regions, with well-defined walls. Each CT finding was recorded as present or absent.

### Lung Biopsy

Thirty-eight patients (12 with acute/subacute ILD and 26 with chronic ILD) underwent surgical lung biopsy. Lung specimens were obtained from at least two sites. Surgical lung biopsy was not performed in patients with severe respiratory failure. Eight patients were autopsied. Sections stained with hematoxylin-eosin were reviewed in all cases. The specimens were categorized on the basis of the following abnormalities consistent with ILD according to the current classification of interstitial pneumonias [19], [20]: usual interstitial pneumonia (UIP), nonspecific interstitial pneumonia (NSIP), bronchiolitis obliterans organizing pneumonia (BOOP), and diffuse alveolar damage (DAD).

### Statistical Analysis

Comparisons between the three groups (PM-ILD, DM-ILD and CADM-ILD) involving binary data was performed either the chi-square test or Fisher’s exact test as appropriate for the sample size. The continuous data from the three groups were compared using the Kruskal-Wallis test. When this was significant, each paring was examined using the Mann-Whitney U test. Survival time was defined as time from initial clinic visit to death (as determined by review of medical records) or censoring. The cumulative survival rate was calculated by using the Kaplan-Meier method. The log-rank test was employed to compare the survival rate between the groups of patients. The Cox proportional hazard model was used for univariate and multivariate analyses. Variables that were significant (p<0.05) predictors in the univariate analysis were included in the multivariate model. All data were expressed as the median with the interquartile range (median, interquartile range). All statistical analyses were performed by using JMP® version 9.0 (SAS Institute, Inc., Cary, NC, USA). A p value of <0.05 was considered indicative of statistical significance.

## Results

### Clinical Features and Laboratory Findings

The clinical characteristics of the patients with myositis-associated ILD (39 men and 75 women; median age, 56 years) are summarized in Table 1. The numbers of patients diagnosed with PM, DM, and CADM were 30, 41, and 43, respectively. The onset of ILD preceded the initial clinical manifestations of PM/DM/CADM in 22 patients, was concomitant with the onset of PM/DM/CADM in 66 patients, and occurred after the onset of PM/DM/CADM in 26 patients. The acute/subacute form of ILD was present in 59 patients (51.8%) and the chronic form in 55 patients (48.2%). The major pulmonary symptoms at the time of diagnosis of ILD were dyspnea (67.9%), cough (64.0%), and fever (51.8%). Chest auscultation revealed fine crackles in 86.0% of the patients. The clinical characteristics of PM-ILD, DM-ILD, and CADM-ILD are compared (Table 1). The frequency of the acute/subacute form was almost identical among patients with PM-ILD, DM-ILD, and CADM-ILD (46.7%, 53.7%, and 53.5%, respectively). The clinical signs were similar among PM-ILD, DM-ILD, and CADM-ILD. The findings of laboratory tests, pulmonary function tests are presented in Table 2. The median serum level of KL-6, a marker of interstitial pneumonia, was elevated. The frequencies of antinuclear antibody and anti-Jo-1 antibody positivity were 32.5% and 19.3%, respectively. Mildly low values of PaO_2_, FVC (%predicted), and %DLCO were observed. The results of the laboratory tests, pulmonary function tests are compared among the PM-ILD, DM-ILD, and CAMD-ILD groups (Table 2). Some differences in the laboratory findings can be seen among the PM-ILD, DM-ILD, and CAMD-ILD groups. The serum creatine phosphokinase (CPK) and aldolase levels were significantly higher in the patients with PM-ILD than in those with DM-ILD or CADM-ILD. However, the results of the pulmonary function tests did not differ significantly among the three groups. The results of BAL findings are presented in Table 3. Analysis of the cellular component of the BAL fluid revealed a relatively high percentage of lymphocytes. No difference in differential cell count in the BAL fluid was observed among the PM-ILD, DM-ILD, and CADM-ILD groups.

**Table 1:** Characteristics of patients with myositis-associated ILD.

	Total	PM-ILD	DM-ILD	CADM-ILD
**Number of patients**	114	30	41	43
**Age, yrs**	56 (49, 65)	54.5 (48, 62)	59 (51.5, 67.5)	56 (49, 64)
**Sex, female**	75 (65.8)	15 (50.0)	28 (68.3)	32 (74.4)
**Time of ILD onset**				
Before PM/DM/CADM onset	22 (19.3)	4 (13.3)	16 (39.0)	2 (4.7)
Concomitant with PM/DM/CADM	66 (57.9)	19 (63.4)	19 (46.4)	28 (65.1)
After PM/DM/CADM onset	26 (22.8)	7 (23.3)	6 (14.6)	13 (30.2)
**Form of ILD**				
Acute/subacute	59 (51.8)	14 (46.7)	22 (53.7)	23 (53.5)
Chronic	55 (48.2)	16 (53.3)	19 (46.3)	20 (46.5)
**Signs**				
Dyspnea during effort	66 (57.9)	17 (56.7)	25 (61.0)	24 (55.8)
Cough	73 (64.0)	22 (73.3)	23 (56.1)	28 (65.1)
Fever	59 (51.8)	17 (56.7)	24 (58.5)	18 (41.9)
Arthralgia	52 (45.6)	15 (50.0)	21 (51.2)	16 (39.0)
**Fine crackles**	98 (86.0)	24 (85.7)	35 (94.6)	39 (95.1)

Data are presented as the medians (interquartile ranges) or n (%).

ILD: interstitial lung disease; PM: polymyositis; DM: dermatomyositis; CADM: clinically amyopathic dermatomyositis.

**Table 2:** Laboratory findings and pulmonary function tests in myositis-associated ILD.

	Total	PM-ILD	DM-ILD	CADM-ILD	P<0.05
**Laboratory findings**					
WBC, mm^−3^	6815 (5253, 9693)	8930 (5993, 12625)	7700 (5350, 13980)	5720 (4700, 7300)	†, ‡
CRP, mg/dL	0.77 (0.16, 2.6)	0.77 (0.2, 3.2)	1.6 (0.3, 3.5)	0.28 (0.1, 1.4)	‡
CPK, IU/L	332 (117, 1166)	1862 (842, 4915)	582 (250, 956)	109 (71, 163)	*, †, ‡
Aldolase, IU/L	12.2 (6.3, 24.6)	26.3(12.4, 49.4)	12.8 (7.9, 26.8)	7.1 (4.3, 12.0)	*, †, ‡
LDH, IU/L	393 (287, 598)	630 (430, 882)	399 (301, 566)	307 (217, 417)	*, †, ‡
IgG, mg/dL	1667 (1365, 2027)	1869 (1463, 2104)	1473 (1243, 2020)	1669 (1518, 1974)	NS
KL-6, U/mL	1115 (728.5,1477.5)	1747 (760, 3196)	1180 (767, 1470)	960 (652, 1367)	†
Positive ANA, %	32.5	23.3	41.5	30.2	NS
Positive Jo-1, %	19.3	31.0	15.4	17.1	NS
PaO_2_, Torr	71.7 (63.4, 80.7)	75.0 (64.3, 86.4)	69.0 (63.0, 80.6)	72.2 (66.2, 80.7)	NS
PaCO_2_, Torr	38 (35.6, 40.7)	39.4 (35.9, 42.7)	37.1 (34.8, 40.0)	38.7 (35.1, 40.7)	NS
**Pulmonary function tests**					
FVC, % predicted	66.3 (56.9, 82.0)	71.1 (57.7, 90.0)	65.4 (57.0, 77.2)	66.4 (54.8, 83.6)	NS
FEV1/FVC, %	83.3 (76.1, 88.3)	85.5 (76.8, 89.6)	83.3 (77.3, 86.2)	82.6 (75.5, 90.4)	NS
%DLCO, %	65.5 (51.7, 82.9)	78.5 (48.1, 89.2)	65.1 (51.0, 81.3)	64.3 (51.3, 84.7)	NS

*; PM vs DM, †; PM vs CADM, ‡; DM vs CADM, NS; not significant in Kruskal-Wallis test.

Data are presented as the medians (interquartile ranges).

ILD: interstitial lung disease; WBC: white blood cell count; CRP: C-reactive protein; CPK: creatine phosphokinase; LDH: lactic dehydrogenase; IgG: immunoglobulin G; ANA: antinuclear antibody; FVC: forced vital capacity; FEV1: forced expiratory volume; DLCO: diffusing capacity of the lung for carbon monoxide.

**Table 3:** Bronchoalveolar lavage (BAL) findings in myositis-associated ILD.

BAL findings	Total	PM-ILD	DM-ILD	CADM-ILD	P value
Number of patients	76	18	29	29	
Macrophages, %	79 (50, 89)	87.3 (71.1, 91.0)	72.7 (48.8, 86.6)	77.8 (52.3, 86.2)	NS
Lymphocytes, %	13.7 (6.3, 31.0)	7.5 (5.0, 18.9)	17.8 (8.0, 45.1)	14.4 (7.2, 30.7)	NS
Neutrophils, %	2.7 (0.4, 6.2)	2.8 (0.2, 5.0)	2.0 (1.1, 8.2)	4.0 (0.2, 6.3)	NS
Eosinophils, %	0.8 (0.2, 2.7)	0.2 (0, 1.5)	1.0 (0.4, 3)	1.2 (0.4, 3.6)	NS
CD4/CD8 ratio	0.59 (0.24, 0.93)	0.44 (0.23, 0.76)	0.49 (0.20, 0.77)	0.7 (0.44, 1.2)	NS

Data are presented as the medians (interquartile ranges).

ILD: interstitial lung disease.

### HRCT and Histological Findings

HRCT images of the lung were available for 102 patients, and the frequency of the findings is summarized in Table 4. Ground glass opacities (88.2%) and traction bronchiectasis (77.5%) were the most frequent findings, followed by consolidation (65.7%) and irregular linear opacities (53.9%). The frequencies of honeycombing and pleural effusion were relatively low (3.9% and 8.8%, respectively). No difference in the HRCT findings was observed among the PM-ILD, DM-ILD, and CADM-ILD groups (Table 4). The histopathological findings in the specimens obtained by surgical lung biopsy (10 PM-ILD, 14 DM-ILD, and 14 CADM-ILD) and autopsy (three DM-ILD and five CADM-ILD) are summarized in Table 5. The most common pathological pattern was NSIP (33 cases, 71.7%). DAD was seen in the eight autopsy specimens, all of which were from patients with the acute/subacute form. UIP was less frequent, appearing in five cases (10.9%).

**Table 4:** High-resolution computed tomography (HRCT) findings in myositis-associated ILD.

HRCT findings (%)	Total	PM-ILD	DM-ILD	CADM-ILD	P value
Number of patients	102	25	40	37	
Consolidation	65.7 (67)	56.0	72.5	64.9	NS
Ground glass opacities	88.2 (90)	96.0	87.5	83.8	NS
Traction bronchiectasis	77.5 (79)	76.0	77.5	78.4	NS
Irregular linear opacities	53.9 (55)	64.0	50.0	51.4	NS
Bronchovascular bundle thickening	39.2 (40)	48.0	40.0	32.4	NS
Honeycombing	3.9 (4)	4.0	2.5	2.7	NS
Pleural effusion	8.8 (9)	4.0	15.0	5.4	NS

Data are presented as the percentage (n) of presence in the findings.

ILD: interstitial lung disease.

**Table 5:** Histological findings in surgical lung biopsy cases and autopsy cases of myositis-associated ILD.

Histological findings	Total	PM-ILD	DM-ILD	CADM-ILD
NSIP	33 (71.7)	9	12	12
UIP	5 (10.9)	1	2	2
DAD	8 (17.4)	0	3	5

Data are presented as the n (%).

ILD: interstitial lung disease; NSIP: nonspecific interstitial pneumonia; UIP: usual interstitial pneumonia; DAD: diffuse alveolar damage.HRCT Findings.

### Treatment and Mortality

In 23 patients, treatment consisted of corticosteroids alone, usually in the form of oral prednisolone (40–60 mg/day) but occasionally as intravenous methylprednisolone pulse therapy (1 g/day for three days). Immunosuppressive agents such as cyclosporine, cyclophosphamide, and/or azathioprine were administered in addition to corticosteroid therapy in 88 patients (Table 6). Intravenous immunoglobulins were administered to 11 patients who did not respond to corticosteroids plus immunosuppressive agents. A total of 31 patients (27.2%) died during the observation period (Table 6). The overall mortality rates among patients with PM-ILD, DM-ILD, and CADM-ILD were 16.7%, 24.4%, and 37.2%, respectively (Table 6). In the 38 cases with surgical lung biopsy (33 NSIP and 5 UIP), three patients with NSIP and one with UIP died during the observation period.

**Table 6:** Treatment and outcome in myositis-associated ILD.

Treatment	Total	PM-ILD	DM-ILD	CADM-ILD
Number of patients	114	30	41	43
Corticosteroids alone	23	9	7	7
Corticosteroids + immunosuppressive agents	88	21	33	34
Cyclosporine	75	12	31	32
Cyclophosphamide	22	5	7	10
Azathioprine	13	8	3	2
Intravenous Igs	11	1	5	5
Mortality (%)	31 (27.2)	5 (16.7)	10 (24.4)	16 (37.2)

Data are presented as the n (%).

ILD: interstitial lung disease; Ig: immunoglobulin.

### Overall Survival Analysis

**Figure 1:**
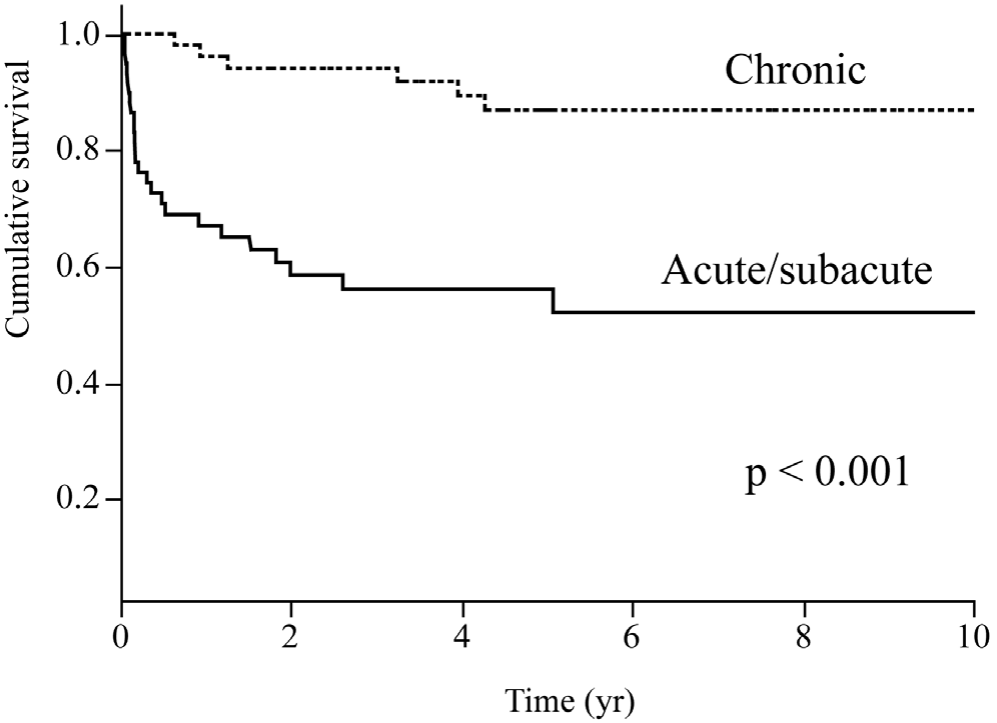
Survival curves for the acute/subacute and chronic forms of ILD in patients with PM/DM/CADM. Patients with the acute/subacute form have a significantly lower survival rate than those with the chronic form (log-rank, p<0.0001).

**Figure 2:**
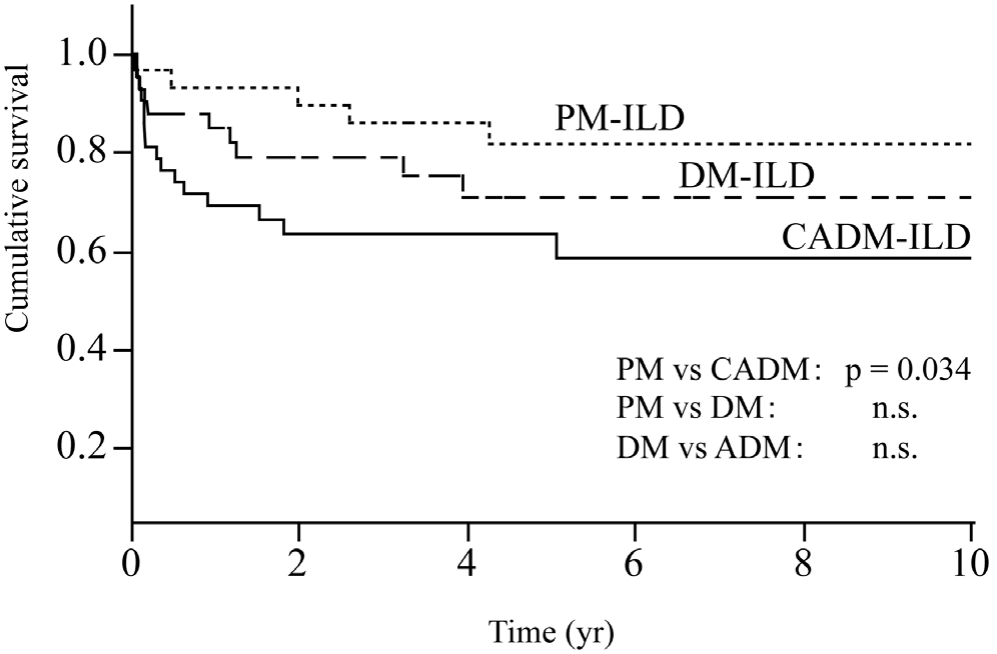
Survival curves of patients with PM-ILD, DM-ILD and CADM-ILD. Patients with CADM-ILD have a significantly lower survival rate than those of PM-ILD (log-rank, p = 0.0034).

The results of the univariate Cox proportional hazards models are shown in Table 7. Acute/subacute form, %FVC, age, % of neutrophils in BAL fluid, and a diagnosis of CADM (vs. PM) were significantly associated with a worse prognosis. None of the HRCT findings was associated with poor outcomes. In the 38 cases with surgical lung biopsy, univariate Cox proportional hazards models analysis revealed that pathological patterns (UIP or NSIP) were not associated with a worse prognosis (UIP vs NISP, hazard ratio 1.944, p = 0.588). Multivariate Cox proportional hazards model analysis revealed that acute/subacute form, age, %FVC, and a diagnosis of CADM (vs. PM) were independent predictors of overall mortality (Table 8). Comparison of the survival rate between the acute/subacute and chronic forms is shown in Figure 1. Patients with the acute/subacute form of ILD had a significantly lower survival rate than did those with the chronic form (five-year survival, 52% vs. 87%, respectively; p<0.0001). The survival curves for patients with PM-ILD, DM-ILD, and CADM-ILD are shown in Figure 2. The survival rate was significantly lower in the patients with CADM-ILD than in those with PM-ILD (p = 0.034). The survival analysis suggested that the outcome in patients with DM-ILD was worse than that in patients with PM-ILD but better than that in patients with CADM-ILD (five-year survival: PM-ILD, 82%; DM-ILD, 71%; CADM-ILD, 59%), although no significant differences in survival rate were found between PM-ILD and DM-ILD or between DM-ILD and CADM-ILD.

**Table 7:** Overall survival analysis in myositis-associated ILD (Cox proportional hazards model, univariate).

	Hazard ratio	95% CI	P value
Age, yrs	1.046	1.012–1.084	0.007
Female sex	1.122	0.5417–2.489	0.763
CVD diagnosis			
DM vs PM	1.771	0.6274–5.670	0.286
CADM vs PM	2.854	1.115–8.746	0.028
CADM vs DM	1.612	0.7413–3.679	0.230
Acute/subacute form	5.477	2.389–14.80	<0.001
LDH (IU/L)	0.9999	0.9992–1.000	0.776
CPK (IU/L)	0.9997	0.9993–1.000	0.059
Aldolase (IU/L)	0.9915	0.9655–1.008	0.369
CRP (mg/dL)	1.109	0.9994–1.210	0.051
KL-6 (U/mL)	0.9999	0.9994–1.0003	0.884
Positive Jo-1	0.6016	0.1771–1.551	0.316
%FVC	0.9612	0.9345–0.9866	0.002
BAL findings			
Lymphocytes, %	0.9981	0.9727–1.019	0.875
Neutrophils, %	1.045	1.010–1.073	0.016
Eosinophils, %	0.9604	0.7810–1.085	0.586
HRCT findings			
Consolidation	1.468	0.5735–4.496	0.441
Ground glass opacities	1.300	0.2715–23.31	0.790
Traction bronchiectasis	0.819	0.2374–5.141	0.794
Irregular linear opacities	1.316	0.5465–3.477	0.548
Bronchovascular bundle thickening	1.028	0.4137–2.487	0.952
Pleural effusion	1.135	0.1797–3.965	0.868

ILD: interstitial lung disease; BAL: bronchoalveolar lavage; HRCT: high-resolution computed tomography. Hazard ratio of honeycombing could not be caluculated, because none of the patients with honeycombing died during observation period.

**Table 8:** Overall survival analysis in myositis-associated ILD (Cox proportional hazards model, multivariate).

	Hazard ratio	95% CI	P value
Age, yrs	1.059	1.019–1.102	0.003
CVD diagnosis			
DM vs PM	2.186	0.6223–10.10	0.231
CADM vs PM	4.184	1.316–18.53	0.014
CADM vs DM	1.913	0.7933–4.895	0.149
Acute/subacute form	4.233	1.690–12.09	0.002
%FVC	0.9603	0.9277–0.9902	0.008

ILD: interstitial lung disease; 95% CI: 95% confidence interval; CVD: collagen vascular disease; PM: polymyositis; DM: dermatomyositis; CADM: clinically amyopathic dermatomyositis; FVC: forced vital capacity.

## Discussion

In the present study, the authors retrospectively reviewed 114 consecutive cases of PM/DM/CADM-ILD and attempted to elucidate their clinical features and prognostic factors. Our findings demonstrated the form of ILD onset, %FVC, age, % of neutrophils in the BAL fluid, and a diagnosis of CADM (vs. PM) to be significant predictors of survival in univariate Cox proportional hazards models. Moreover, multivariate Cox proportional hazards model analysis revealed the acute/subacute form of ILD, older age, lower FVC, and a diagnosis of CADM (vs. PM) to be independent predictors of poor prognosis in patients with PM/DM/CADM-ILD. Only a few studies thus far have assessed the prognostic factors for PM/DM-ILD [8], [14]. To the best of our knowledge, the present retrospective study included the largest cohort of PM/DM/CADM patients with ILD (114 consecutive cases) to date.

We found that the acute/subacute form of ILD was a strong predictor of poor outcome in patients with PM/DM/CADM-ILD. Most cases of ILD associated with collagen vascular diseases (CVD) follow a chronic indolent course; however, acute progression of ILD has been reported in patients with PM, DM, and CADM [7], [11], [21], [22]. Won Huh *et al.* reported that one-third of patients with PM/DM-ILD exhibited acute progression and that the three-year survival rate was lower for the acute form (27.3%) than for the chronic form (78.8%) [21]. These findings suggest that acute progression of myositis-associated ILD may be associated with poor prognosis; however, this required verification by using multivariate analysis in a large cohort of patients with PM, DM, and CADM. The present study was the first to identify the acute/subacute form as an independent prognostic factor for PM/DM/CADM-ILD by using a multivariate Cox proportional hazards model. The five-year survival rate was much lower for the acute/subacute form than for the chronic form (52% vs. 87%, respectively). Although the clinical characteristics of myositis-associated ILD may differ in each case, it should be noted that the acute/subacute form carries a poor prognosis regardless of the specific diagnosis (PM, DM, or CADM) and may require intensive treatment.

In terms of prognosis of myositis-associated ILD, a few studies have highlighted the clinical implications of the specific CVD diagnosis. Recently, Mukae and colleagues [22] reported differences in clinical characteristics and outcomes between 16 patients with DM-ILD and 11 with CADM-ILD. Their findings demonstrated that the higher prevalence of the acute subtype of ILD resulted in a higher mortality rate in patients with CADM-ILD than in those with DM-ILD. In the present study of a large number of consecutive patients with PM/DM/CADM-ILD, we showed that patients with CADM-ILD had significantly worse outcomes than those with PM-ILD. Consistent with the results of previous studies [6], [23], survival analysis suggested that the outcome in patients with DM-ILD was worse than that in patients with PM-ILD but better than that in patients with CADM-ILD (five-year survival: PM-ILD, 82%; DM-ILD, 71%; CADM-ILD, 59%). Considering that the prevalence of acute/subacute form was almost identical among patients with PM-ILD, DM-ILD, and CADM-ILD in the current study (PM-ILD; 46.7%, DM-ILD; 53.7%, CADM-ILD; 54.5%), the difference of survival among the three groups was not merely due to the prevalence of acute/subacute form. Moreover, multivariate analysis revealed that both diagnosis of CADM and the acute/subacute form of ILD were independently associated with poor outcome. Differences in the pathological mechanisms of PM-ILD, DM-ILD, and CADM-ILD may account for the variation in mortality.

There have been few published investigations of the prognostic factors for ILD associated with PM/DM. Marie and colleagues [8] reviewed 107 patients with PM/DM-ILD and divided them into two groups, ILD deterioration (17 patients) and absence of ILD deterioration (90 patients), according to the course of ILD. They simply compared these two groups and showed that the patients with ILD deterioration were older and had more symptomatic ILD, lower values of FVC and DLCO, a higher frequency of the UIP pattern according to HRCT and lung biopsy, and a higher mortality rate than those without ILD deterioration; however, their findings did not clarify the prognostic factors among all patients with PM/DM-ILD. Our analysis using a multivariate Cox proportional hazards model verifies that the acute/subacute form of ILD, older age, lower %FVC, and a diagnosis of CADM can predict poor outcome in patients with PM/DM/CADM-ILD. Analysis of the histological findings in our 38 cases in which surgical lung biopsy was performed revealed that NSIP was a much more common histologic pattern than was UIP. In addition, neither the UIP pattern nor the NSIP pattern was associated with worse survival in the 38 cases with surgical lung biopsy. All eight autopsy specimens analyzed came from patients with the acute/subacute form of ILD and exhibited DAD, indicating that DAD may be a characteristic finding of the acute/subacute form of ILD and also a poor prognostic indicator. Recently, anti-CADM-140 antibody (also referred to as anti-melanoma differentiation-associated gene 5 (anti-MDA5) antibody) was found to be myositis-specific [24]. Anti-MDA5 antibody was detected in patients with CADM [24], and presence of anti-MDA5 antibody was associated with rapidly progressive ILD and poor outcome [14], [25]–[27]. We did not examine the presence of anti-MDA5 antibody in the present study because no assay for measurement of anti-MDA5 antibody was commercially available. In the future, examination for anti-MDA5 antibody should be considered in the management of patients with myositis-associated ILD.

The present study has certain limitations. First, it was a retrospective study based on patients seen at pulmonary clinics. It is likely that the patients seen at these centers represent a population with more advanced lung involvement, which may have caused selection bias and relatively high mortality and frequency of the acute/subacute form compared with the patients with PM/DM-ILD in another recently published study [21]. Second, the patients included in the present study were not treated according to a consistent plan. Most of the patients were treated with corticosteroids with additional immunosuppressive agents; however, variations in the therapeutic regimen may have affected the responses to therapy and outcomes. Third, it is possible that muscular manifestations and extra-muscular manifestations (e.g. cutaneous lesions) except for ILD could have influenced mortality in the patients with PM/DM/CADM-ILD. We may not be aware of some manifestations that were encountered during clinical courses. Koga and colleagues [26] reported that the DM/CADM patients with anti-MDA5 antibody had higher prevalence of skin ulcer and palmar papules and worse prognosis than those without anti-MDA5 antibody. Despite these limitations, the present large cohort study can provide significant information about prognostic features of PM/DM/CADM-ILD. Given the rarity of PM/DM/CADM-ILD, it is unlikely that a randomized trial will be performed soon.

In summary, the present study demonstrated that the acute/subacute form of ILD, older age, lower %FVC, and a diagnosis of CADM are independent predictors of poor outcome of PM/DM/CADM-ILD. Survival analysis showed significantly higher mortality in patients with acute/subacute ILD than in those with chronic ILD. Patients with CADM-ILD had worse outcome than those with PM-ILD. These factors should be considered in order to provide appropriate management for patients with PM/DM/CADM-ILD. Further studies will address the optimal treatment for patients with PM/DM/CAMD-associated ILD.
